# Draft genomes of *Dehalococcoides mccartyi* strain J1 and *Trichlorobacter lovleyi* strain J2 from a tetrachloroethene-dechlorinating consortium

**DOI:** 10.1128/mra.00485-25

**Published:** 2025-07-01

**Authors:** Huijuan Jin, Xiuying Li, Jingjing Wang, Yiru Cui, Siqi Huang, Ke Shi, Tongyue Zhou, Jun Yan

**Affiliations:** 1 Key Laboratory of Pollution Ecology and Environmental Engineering, Institute of Applied Ecology, Chinese Academy of Sciences 74763 , Shenyang, Liaoning, China; 2 University of the Chinese Academy of Sciences 74519, Beijing, China; Montana State University, Bozeman, Montana, USA

**Keywords:** *Dehalococcoides mccartyi*, *Trichlorobacter lovleyi*, reductive dechlorination, tetrachloroethene, ethene

## Abstract

Two metagenome-assembled genomes were recovered from an anaerobic consortium capable of complete reductive dechlorination of tetrachloroethene to ethene. The draft genomes, assigned to *Dehalococcoides mccartyi* strain J1 and *Trichlorobacter lovleyi* strain J2, are 1.37 and 3.84 Mb in size and encode 1,448 and 3,630 genes, respectively.

## ANNOUNCEMENT

*Dehalococcoides mccartyi* and *Trichlorobacter lovleyi* are keystone organohalide-respiring bacteria (OHRB) that play crucial roles in the cleanup of sites contaminated with chlorinated solvents, such as tetrachloroethene (PCE) (1–3). We collected freshwater sediment from a location (41.6628°N, 123.1055°E) at the Xi River (Shenyang, Liaoning, China) (4) as the source material to explore the diversity and environmental distributions of *D. mccartyi* and *T. lovleyi*. Anaerobic microcosm was established in sealed 160 mL serum bottles by inoculating approximately 2 g sediment into 100 mL reduced, bicarbonate-buffered mineral medium (5) containing 0.5 mM PCE (aqueous phase concentration), 10 mL H_2_, 10 mM lactate, an 80:20 N_2_/CO_2_ headspace, and incubated statically at 30°C for 1 month. Repeated transfers (1%–3%, vol/vol) under the same growth conditions yielded a solid-free consortium able to sequentially dechlorinate PCE to ethene with the transient accumulation of several less-chlorinated ethenes (e.g., vinyl chloride), as analyzed using a GC-FID system (6).

Metagenomic sequencing was conducted to investigate the composition and genomic features of the OHRB populations in the PCE-dechlorinating community. Cells at the stationary phase from 1.6 L culture were harvested by centrifugation, and genomic DNA was extracted following JGI’s CTAB protocol. DNA quantity and purity were measured using a NanoDrop One spectrophotometer (Thermo Fisher Scientific, Waltham, MA, USA). Prior to Illumina HiSeq 2500 sequencing, libraries were prepared with the VAHTS Universal Plus DNA Library Prep kit (Vazyme Biotech, Nanjing, China) following the manufacturer’s instructions and assessed using an Agilent 2100 Bioanalyzer (Agilent Technologies, Santa Clara, CA, USA). The obtained 120,326,154 paired-end reads (2 × 150 bp) were quality-filtered with Trimmomatic version 0.36 and assembled using metaSPAdes version 3.15.3 (7, 8). Binning was performed using MaxBin2 version 2.2.4, taxonomic classification was carried out with GTDB-Tk version 2.3.2, and genome quality was assessed using CheckM version 1.0.18 (9–11). Two metagenome-assembled genomes, representing a *D. mccartyi* strain, designated as J1, and a *T. lovleyi* strain, designated as J2, were annotated with NCBI Prokaryotic Genome Annotation Pipeline version 6.7 (12). The average nucleotide identity (ANI) values were calculated using JSpeciesWS version 4.1.1 (13). Default settings were used for bioinformatics tools.

The draft genomes of *D. mccartyi* strain J1 and *T. lovleyi* strain J2 consist of 3 and 47 contigs, respectively, with their genomic features summarized in Table 1. Comparative genomic analysis revealed that strain J1 is most closely related to *D. mccartyi* strain CG4 (14), sharing 97.8% ANI and 100% 16S rRNA gene sequence identity. Similarly, strain J2 showed high similarity to *T. lovleyi* strain SZ (1), with 97.9% ANI and 99.7% 16S rRNA gene identity. Twelve and two putative reductive dehalogenase (RDase) genes were annotated in strain J1 and strain J2 genomes, respectively. An inferred RDase of strain J1 shares 99.6% amino acid identity with the TceA, which catalyzes the reductive dechlorination of dichloroethenes and vinyl chloride to ethene. Both RDase genes identified in strain J2 are homologous to the well-characterized *pceA*, evidencing this population’s involvement in PCE reductive dechlorination. Taken together, the basic features of recovered *D. mccartyi* and *T. lovleyi* genomes align with the observed PCE-dechlorinating and ethene-forming capability in this sediment-derived consortium.

**TABLE 1:** Basic genomic features of *D. mccartyi* strain J1 and *T. lovleyi* strain J2

	*D. mccartyi* strain J1	*T. lovleyi* strain J2
No. of contigs	3	47
*N*_50_	868,288	155,865
*L*_50_	1	8
GC content (%)	48.8	55.0
Size (bp)	1,374,970	3,838,454
Genome coverage	58.3×	72.6×
Completeness (%)	99.0	99.7
Contamination (%)	0.0	1.3
Number of RNAs	50	45
Number of identified genes	1,448	3,630
Number of identified CDSs	1,398	3,585
GenBank accession no.	JBNLRZ000000000	JBNLSA000000000

## Data Availability

The draft genomes of *D. mccartyi* strain J1 and *T. lovleyi* J2 have been deposited at GenBank under accession numbers JBNLRZ000000000 and JBNLSA000000000. The BioSample and BioProject accession numbers are SAMN48157843 and PRJNA1255881 for *D. mccartyi* strain J1, SAMN48157844 and PRJNA1255882 for *T. lovleyi* strain J2, respectively. Metagenome sequencing raw reads were deposited in the Sequence Read Archive (SRA) under accession number SRR32383007.
